# Genomic and transcriptomic insights into *Raffaelea lauricola* pathogenesis

**DOI:** 10.1186/s12864-020-06988-y

**Published:** 2020-08-20

**Authors:** Yucheng Zhang, Junli Zhang, Dan Vanderpool, Jason A. Smith, Jeffrey A. Rollins

**Affiliations:** 1 Department of Plant Pathology, University of Florida, 1453 Fifield Hall, Gainesville, FL 32611-0680 USA; 2 School of Forest Resources and Conservation, University of Florida, Gainesville, FL 32611-0410 USA; 3 Division of Biological Sciences, University of Montana, Missoula, MT USA; 4 Present address: Department of Biology and Department of Computer Science, Indiana University, 1001 E. 3rd Street, Bloomington, IN 47405 USA

**Keywords:** Laurel wilt, *Raffaelea lauricola*, Genome, Sulfur, Aerolysin, Ceratoplatanin, Transcriptome, Effector, Secondary metabolite, Ophiostomatales, Vascular wilt disease

## Abstract

**Background:**

Laurel wilt caused by *Raffaelea lauricola* is a lethal vascular disease of North American members of the Lauraceae plant family. This fungus and its primary ambrosia beetle vector *Xyleborus glabratus* originated from Asia; however, there is no report of laurel wilt causing widespread mortality on native Lauraceae trees in Asia. To gain insight into why *R. lauricola* is a tree-killing plant pathogen in North America, we generated and compared high quality draft genome assemblies of *R. lauricola* and its closely related non-pathogenic species *R. aguacate.*

**Results:**

Relative to *R. aguacate*, the *R. lauricola* genome uniquely encodes several small-secreted proteins that are associated with virulence in other pathogens and is enriched in secondary metabolite biosynthetic clusters, particularly polyketide synthase (PKS), non-ribosomal peptide synthetase (NRPS) and PKS-NRPS anchored gene clusters. The two species also exhibit significant differences in secreted proteins including CAZymes that are associated with polysaccharide binding including the chitin binding CBM50 (LysM) domain. Transcriptomic comparisons of inoculated redbay trees and in vitro-grown fungal cultures further revealed a number of secreted protein genes, secondary metabolite clusters and alternative sulfur uptake and assimilation pathways that are coordinately up-regulated during infection.

**Conclusions:**

Through these comparative analyses we have identified potential adaptations of *R. lauricola* that may enable it to colonize and cause disease on susceptible hosts. How these adaptations have interacted with co-evolved hosts in Asia, where little to no disease occurs, and non-co-evolved hosts in North America, where lethal wilt occurs, requires additional functional analysis of genes and pathways.

## Background

Emerging infectious diseases of plants and animals due to anthropogenic movement, climate change, and natural processes are impacting natural ecosystems at an unprecedented rate [1–3]. Laurel wilt is among these diseases. The disease is caused by the Ascomycota fungus *Raffaelea lauricola* (Ophiostomatales), a native symbiont of the invasive Asian ambrosia beetle *Xyleborus glabratus* (Curculionidae: Scolytinae). In North America, *X. glabratus* was first detected in Port Wentworth, Georgia, USA in 2002 [4] and the wilting and mortality of native Lauraceae trees in the area were first reported in 2003 [5]. In the ensuing time, laurel wilt has caused and continues to cause widespread mortality on redbay (*Persea borbonia*) and other members of the Lauraceae family in the southeastern USA resulting in massive ecosystem damage [6–11]. In 2011, laurel wilt was found to infect avocado in Florida’s commercial production area [12]. Infection of this domesticated and agronomically important Lauraceae family member has significantly impacted the commercial production of avocado in Florida [13] and poses a serious threat to currently unaffected avocado-producing areas of North America including California and Mexico [14].

The insect vector *X. glabratus* has been recorded from Bangladesh, India, Japan, Myanmar, Taiwan, and mainland China and is thought to be native to Asia [15, 16]. *R. lauricola* has been recovered from specimens of *X. glabratus* from Japan and Taiwan [17]. Despite the widespread occurrence of *X. glabratus* in Asia, there is no report of laurel wilt causing mortality on native Lauraceae trees in Asia. Beetle galleries and limited wilt and vascular streaking symptoms were however reported from native stands of Asian Lauraceae trees in Taiwan [18]. In the southeastern USA where laurel wilt has annihilated native Lauraceae trees, invasive camphor trees from Asia (*Cinnamomum camphora*) exhibit limited symptoms including branch dieback or no symptoms of laurel wilt unless subject to mass beetle attacks [19]. These findings indicate that *R. lauricola* is a pathogen in Asia but not a tree-killing pathogen of native Lauraceae hosts from this native range.

*R. lauricola* in the USA has been assumed to be from a single introduction [17]. This was confirmed by a recent genetic variation analysis of *R. lauricola* populations in Taiwan, Japan, and the USA [20]. This study identified high genetic diversity and both mating types (MAT1 and MAT2) in populations from Taiwan and Japan. On the contrary only MAT2 is found in the USA and is currently represented by a clonally reproducing, highly uniform population based on SSR comparisons of 57 isolates from all known hosts throughout the geographical range [21]. Dreaden et al. [22] have examined North American and Asian isolates and further narrowed the origin of the North American population to Taiwan. Artificial inoculation of *R. lauricola* isolates from Asia kill avocado and swamp bay trees with similar aggressiveness and symptomology as isolates from the USA [23, 24]. These findings indicate that the pathogen lineage introduced to North America is similar in virulence as isolates from the native range.

Like other vascular tree diseases including Dutch elm disease of Wych elm caused by *Ophiostoma novo-ulmi* [25] and *Verticillium* wilts of numerous tree species [26], diseased Lauraceae plants exhibit wilt symptoms including rapid foliage necrosis and vascular discoloration. These symptoms appear to be at least partially related to the xylem blockage caused by tylose and gel formation [27, 28]. *R. lauricola* is so aggressive that a single inoculation of this pathogen on avocado and other members of the North American Lauraceae family is sufficient to induce systemic and lethal disease development within several weeks [27, 29] with as few as 100 spores [30]. The mechanisms underlying this extreme aggressiveness remain uncharacterized.

The mortality of Lauraceae trees in the southeastern USA is at least partially due to an extreme host response [28]. Plant immunity systems functioning against pathogen attack consist of at least two interconnected pathways, namely pathogen-associated molecular pattern (PAMP)-triggered immunity (PTI) and effector-triggered-immunity (ETI) [31]. PTI provides basal defense against all potential pathogens and is based on the recognition of conserved PAMPs by pattern recognition receptors (PRRs) that activate PTI. Chitin, a major structural component of fungal cell walls, is one of the best-characterized fungal PAMPs. If basal PTI defense systems are evaded by pathogen effectors, plants perceive effectors and activate ETI, which leads to rapid and enhanced host defense responses, including hypersensitive responses (HR). Whether PTI or ETI plays the major role in the extreme host response of Lauraceae plants in the southeastern USA to *R. lauricola* is unknown, and various hypotheses have been proposed to explain the extreme symptomology of laurel wilt on North American species of Lauraceae [18, 32, 33]. The first of these hypotheses that we term the “accidental pathogen hypothesis” was originally proposed by Hulcr and Dunn [32] as an example of an “evolutionary mismatch hypothesis”. It proposes that *R. lauricola* is a non-pathogen in trees native to southeast Asia and the lethal symptomology observed in Lauraceae hosts native to the western hemisphere is the result of a massive defense response of the previously un-encountered host species sensing the presence of a potential pathogen within the xylem tissue associated with beetle galleries. Hulcr et al. provided strong evidence against this hypothesis when they reported the presence of mild, i.e., non-lethal, laurel wilt symptomology in Taiwan on Asian species of Lauraceae [18]. A second hypothesis which is truly an “evolutionary mismatch hypothesis” in the context of Desurmont et al. [34] is based on co-evolutionary processes [33] and we term the “adapted pathogen hypothesis”. Under this hypothesis, *R. lauricola* has evolved pathogenicity in its native range via host-pathogen co-evolutionary processes, and a more balanced host response dampens symptomology in extant Lauraceae from the eastern hemisphere. The lack of these co-evolutionary processes from the host defense side results in lethal symptomology in western hemisphere hosts either as an overreaction to PAMPS produced by the pathogen or as the result of specific virulence factors or effectors to which the North American hosts are unadapted. To test and distinguish between these two hypotheses and better understand the adaptations present in pathogenic relative to non-pathogenic *Raffaelea* spp., we generated, annotated, and compared high-quality draft genome assemblies of the pathogenic *R. lauricola* (isolate RL4) and a closely related non-pathogenic species *R. aguacate* (isolate PL1004), recovered from a dead avocado tree (*Persea americana*) in Florida [35]. *R. aguacate* PL1004 resembles *R. lauricola* morphologically, is phylogenetically closely related to *R. lauricola* [36], is a nutritional symbiont of ambrosia beetles that transmit *R. lauricola* and associates with avocado but is not pathogenic. Comparative genomic analysis between *R. lauricola* RL4 and *R. aguacate* PL1004 has the potential to reveal shared and unique genes between the two species and determine if an enrichment for pathogenicity-associated genes exists within *R. lauricola* consistent with the hypothesis of adapted pathogenesis.

## Results

### *Raffaelea lauricola* and *R. aguacate* genome assemblies and gene prediction

To conduct comparative genomics analyses between pathogenic and non-pathogenic *Raffaelea* spp., we sequenced and assembled genomes and transcriptomes of the pathogenic species *R. lauricola* RL4 and the non-pathogenic species *R. aguacate* PL1004 using the pipeline shown schematically in Supplemental Figure S1. Each species was sequenced by Ion Torrent technology to generate 1402 Mb of Q20 bases with average read lengths of 300 bp and 1669 Mb of Q20 bases with average read lengths of 308 bp, respectively. The two species were also previously sequenced [37] from mate-pair and paired-end libraries generated by Illumina Hi-Seq 2000 and assembled (GenBank assembly accession: GCA_002778145.1 and GenBank assembly accession: GCA_002777955.1) by ALLPATHS-LG [38]. To leverage genomic information from both the Illumina and Ion Torrent assemblies, the Metassembler pipeline [39] was utilized. The integrated assemblies generated by Metassembler were improved as evidenced by an increase in N50 and a decrease in the number of scaffolds (Table 1) compared with assemblies using sequence reads from IonTorrent or Illumina reads alone [37, 40]. The non-pathogenic *R. aguacate* PL1004 genome assembly (35.7 Mb) is slightly larger but similar in size to the closely related pathogenic species *R. lauricola* RL4 (34.3 Mb).

**Table 1:** Genome assembly and structural annotation of two *Raffaelea* genomes

Organism	***R. lauricola***	***R. aguacate***
Isolate name	RL4	PL1004
Region of isolation	Florida (Brevard Co.), USA	Florida (Miami-Dade Co.), USA
Sequencing platform	Illumina & Ion torrent	Illumina & Ion torrent
Assembled genome size (Mb)	34.3	35.7
Contig count	480	843
Scaffold count	169	368
Contig N50 (Kb)	394.3	134.8
Scaffold N50 (Kb)	3109.7	458.8
Coding genes	10,315	11,654
Number of complete BUSCOs*	1415 (98.4%)	1406 (97.8%)
Number of Fragmented BUSCOs	21	29
GC content (%)	55.3%	57.5%
Repeat rate (%)	8.64%	6.51%
Reference	This study	This study

*n = 1438

To determine if differences existed in repeat content between pathogenic and non-pathogenic species, we examined and compared repetitive sequences between the two genomes. A total of 8.64% of the *R. lauricola* assembly was identified as repetitive compared with 6.51% for the non-pathogenic *R. aguacate*. The increased repeat content of the *R. lauricola* genome could be attributed primarily to an increase in LTR retroelements which comprised 2% of the *R. lauricola* genome and only 0.2% of the *R. aguacate* genome. LINE retroelements and DNA transposons comprised 0.6 and 1% of the *R. lauricola* genome respectively representing and enrichment of 3-fold and 2-fold over *R. aguacate*. (Table S1).

RNA-Seq data for gene prediction was generated from liquid-grown *R. lauricola* RL4 and *R. aguacate* PL1004 samples with Illumina Hiseq 2000 technology (NCBI SRA Sample accession: SRX3033598 and SRX3033591). To create a comprehensive transcriptome database, we used a pipeline that combined genome-guided and de novo Trinity assemblies [41], followed by Program to Assemble Spliced Alignments (PASA0 [42] to assemble the RNA-Seq reads. These processes generated 25,044 and 26,386 transcripts for *R. lauricola* RL4 and *R. aguacate* PL1004, respectively.

Gene predictions for the two *Raffaelea* genomes were made using the MAKER annotation pipeline [43]. MAKER predicts proteins based on RNA-Seq transcripts and homology with protein-coding sequences of other species, and with the consensus of the ab initio gene prediction algorithms GeneMark [44], AUGUSTUS [45], and SNAP [46]. The details of the Maker pipeline can be found in the Methods section. Using these methods, the *R. lauricola genome* was predicted to encode 10,315 proteins whereas *R. aguacate* was predicted to encode 11,654 proteins. This represents a 13% increase in gene coding capacity in the non-pathogen whose genome is only 4% larger than that of *R. lauricola*. This, coupled with a decreased repeat content, indicates a slightly more streamlined genome for *R. aguacate* relative to *R. lauricola*.

Benchmarking Universal Single-Copy Orthologs (BUSCO) was used to provide an estimate of assembly and annotation completeness [47]. A search for the 1438 fungal universal single-copy ortholog genes with BUSCO 1.2 identified 1415 (98.4%) complete and 21 partial genes in *R. lauricola* RL4 and 1406 (97.8%) complete and 29 partial genes in *R. aguacate* PL1004. Predicted gene content for each genome assembly is therefore estimated to be ~ 98% complete. These figures indicate high quality draft genome assemblies and gene predictions for both *Raffaelea* species. These gene predictions were utilized for further comparative analyses.

#### Secreted protein and candidate effector analysis

Effector proteins play a fundamental role in establishing host-pathogen compatibility as well as in triggering host defense responses. To identify candidate effector proteins from *R. lauricola*, secreted proteins lacking transmembrane domains were analyzed with the EffectorP algorithm [48]. From *R. lauricola*, 49 of the 740 secreted proteins are predicted to be effectors (6.6%; Table S2). From *R. aguacate*, 30 of the 727 secreted proteins are predicted to be effectors (4.1%; Table S3). Of the 49 predicted effectors from *R. lauricola*, five are significantly up-regulated *in planta* (FDR < 0.05) (Table S2) but only one, RL4_JR_10338, a putative glycosyltransferase is unique to *R. lauricola* relative to *R. aguacate*. The other four predicted effectors that were up-regulated during infection share various homologies. RL4_JR_03519 (FDR = 3E-6; 3.1 log2 fold change) has conserved sequence and structural homology with the cysteine-rich secretory protein SCP domain (plant PR-1 family). RL4_JR_05948 (FDR = 1E-4; 3.3 log2 fold change) is highly conserved within fungi and shares homology with ribosomal protein s17 and many hypothetical proteins. RL4_JR_6769 (FDR = 3E-24; 4.6 log2 fold change) encodes a small (67 amino acid) pre-protein with homology only to other hypothetical proteins. RL4_JR_9102 (FDR = 9E-15; 2.3 log2 fold change) encodes a protein with strong homology to peptide methionine sulfoxide reductases which play a role in protecting proteins from oxidative damage. The remaining 44 putative effectors genes encode proteins of diverse putative functions eight of which are significantly down-regulated during infection and 36 were not significantly different in their expression levels during growth in culture versus infected redbay trees (Table S2). Of these 36 genes, 16 are hypothetical proteins, six of which lack orthologs in *R.* aguacate. Another eight are lineage restricted lacking significant homology within the non-redundant protein database and one, common to both *R. lauricola* (RL4_JR_08653) and *R. aguacate* (Rsp272_RL_08445), encodes a necrosis inducing protein (NPP1).

**Fig. 1:**
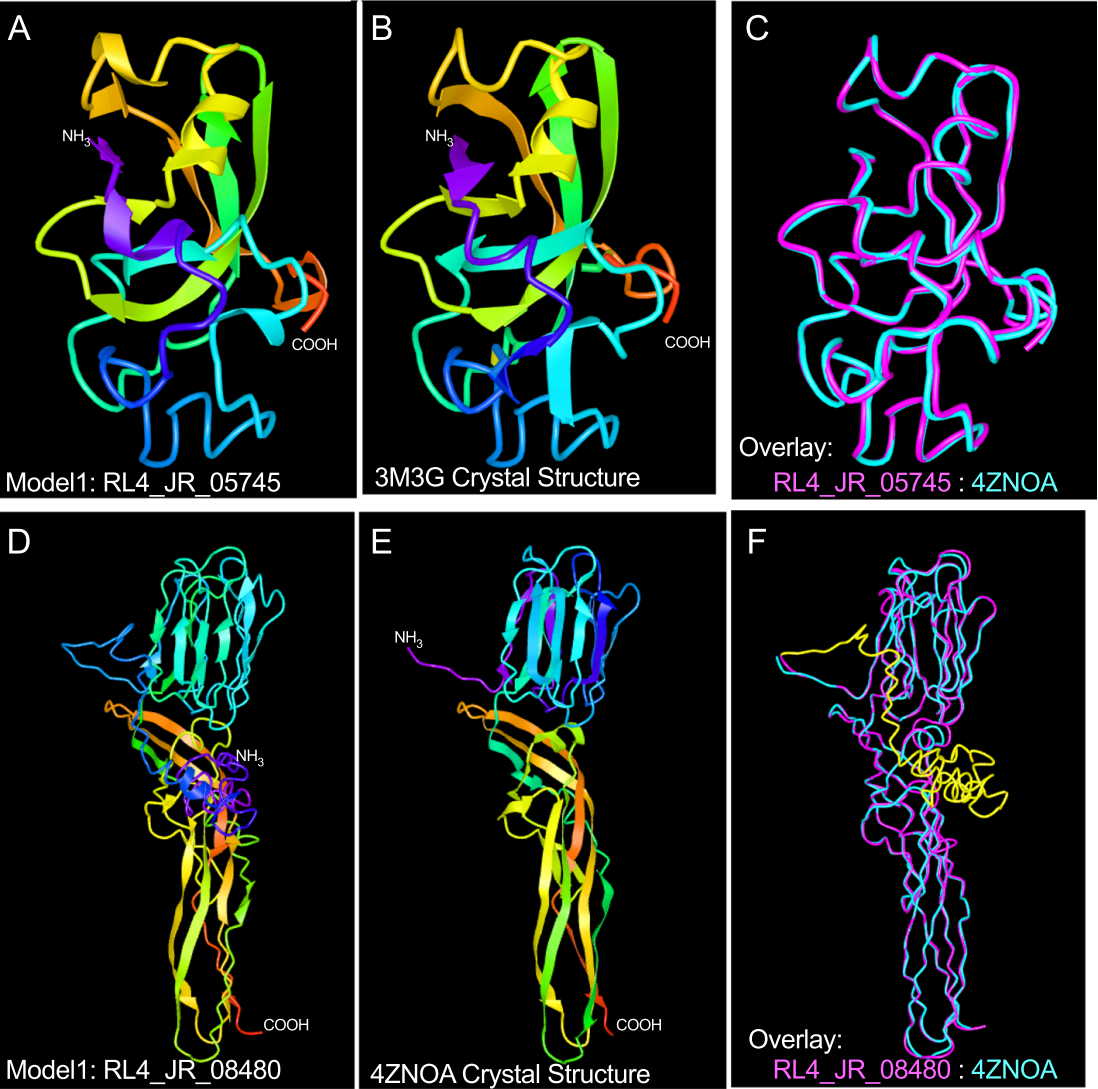
Tertiary protein structure comparisons. **a** Predicted tertiary structure model of the RL4_JR_05745 protein. **b** Tertiary structure of protein 3M3G (*Hypocrea virens*, Sm1:elicitor of plant defense responses), the best Protein Data Bank (PDB) structural match to RL4_JR_05745. **c** Structural alignment of RL4_JR_5745 (magenta) and 3M3G (cyan). **d** Predicted tertiary structure model of the full length RL4_JR_08480 protein. **e** Tertiary structure of protein 4ZNOA (Dln1), the best Protein Data Bank (PDB) structural match to RL4_JR_08480. **f** Structural alignment of RL4_JR_08480 (magenta) and 4ZNOA (Dln1) (cyan), unaligned N-terminus, including the signal peptide, of RL4_JR_08480 (yellow)

Given the relative bias for effector prediction based on known effectors [48] and with the knowledge that the emergence of pathogenic diversity is frequently associated with the gain and loss of genes resulting in a discontinuous taxonomic distribution of effector genes [49, 50] we next examined the novel gene content of *R. lauricola* relative to *R. aguacate*. Between the two genomes, 8128 putatively orthologous pairs were identified representing 79 and 70% of the predicted proteomes of *R. lauricola* and *R. aguacate* respectively. From the 2195 *R. lauricola* unique proteins, 199 are predicted to be secreted (Supplemental Table S4). From the 3529 *R. aguacate* unique proteins, 204 are predicted to be secreted (Supplemental Table S5). Among the unique secreted proteins from *R. lauricola*, several with previously defined roles in fungal pathogenesis and defense elicitation were identified. Prominent among these is a predicted protein (RL4_JR_05745) belonging to the cerato-platanin family. This family consists of small, secreted proteins unique to fungi [51]. This *R. lauricola* cerato-platanin gene shares significant homology to many characterized and predicted cerato-platanin proteins from fungi including homologs in *Grosmannia clavigera* (1E-65) *and Ophiostoma piceae* (1E-61). It shares a 62% identity at the amino acid level with that of BcSpl1, the *Botrytis cinerea* cerato-platanin protein which has been demonstrated to contribute to virulence and elicit a hypersensitive response in its hosts [52, 53]. This protein also possess the typical structural features of this family, including a high percentage (> 40%) of hydrophobic amino acid residues and four conserved cysteine residues (Supplemental Figure S2) and is up-regulated (FDR = 0.01; 1.3 log2 fold change) during plant infection. In addition to its primary sequence homology, RL4_JR_05745 also shares strong tertiary structure similarity to cerato-platanins of other fungi including the founding member of this family from *Ceratocystis platani* (PDB:2KQA_A). The highest scoring (TM-score = 0.987) of these structural homologs is the Sm1 cerato-platanin family member from *Hypocrea virens* (PDB:3M3A) which has been characterized as an elicitor of plant defense responses [54]. The three dimensional model of the predicted RL4_JR_05745 protein and the crystal structure model of Sm1 (3M3A) are shown in Fig. 1a and b, respectively. The overlay of the Sm1 crystal structure and the RL4_JR_05745 model predicts that the proteins share nearly identical tertiary structure (Fig. 1 c) despite a primary sequence identity of only 71%.

**Fig. 2:**
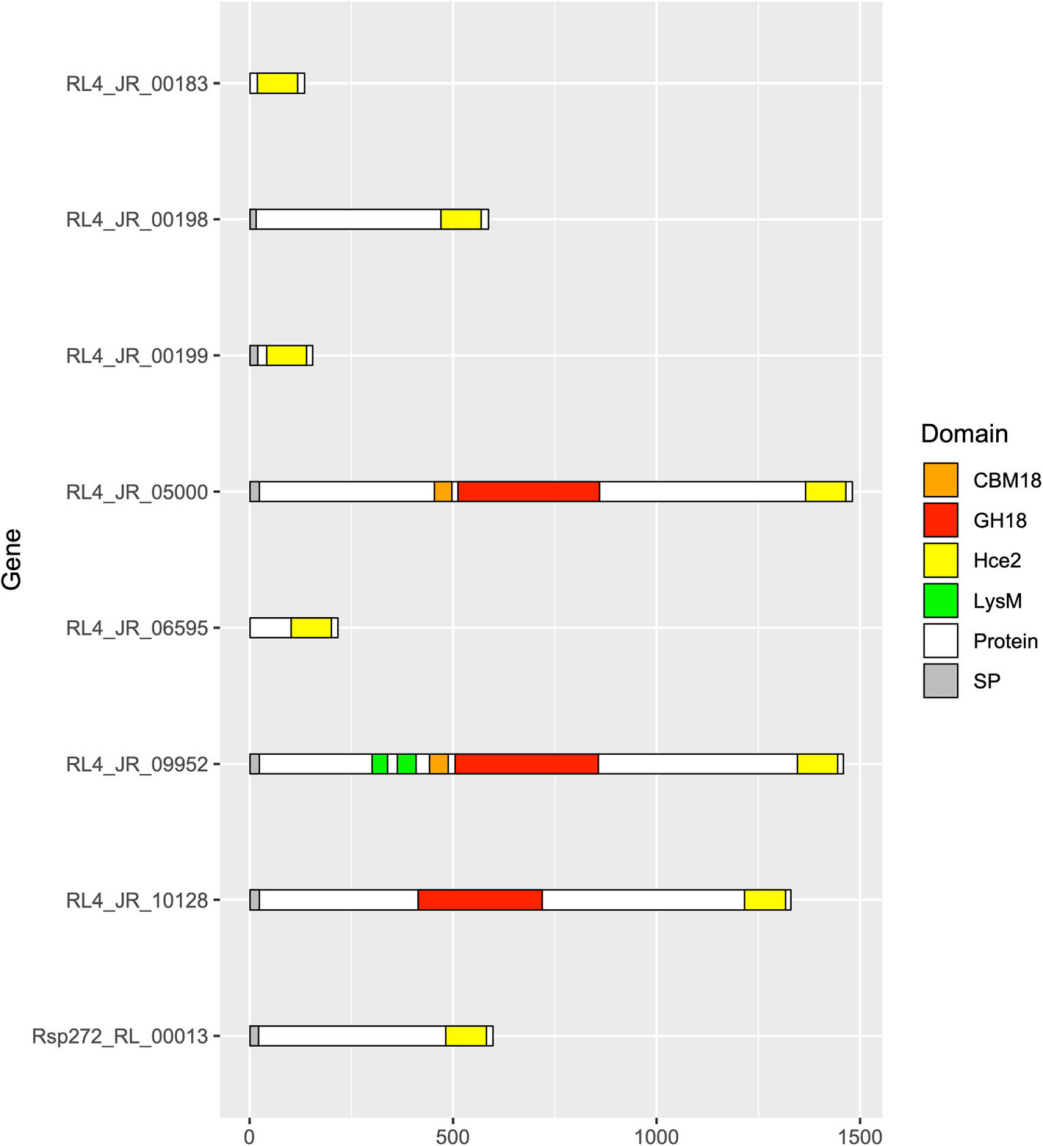
Domain architecture of *Raffaelea lauricola* and *R. aguacate* proteins containing Hce2 domains. CBM18: chitin binding 1 domain (PF00187); GH18: chitinase (PF00187); Hce2: putative necrosis-inducing factor (PF14856); LysM (CBM50): chitin binding (PF01476); SP: signal peptide

Another putative effector not predicted by EffectorP but found to be unique to *R. lauricola* relative to *R. aguacate* is a member of the Hce2 family homologous to the *Cladosporium fulvum* (*Passalora fulvum*) Ecp2 [55]. In total, *R. lauricola* contains seven *hce2* genes and *R. aguacate* possesses one (Fig. 2). Among these, only one *R. lauricola* protein, RL4_JR_00199, a predicted 155 aa secreted protein, matches the structural characteristics common to extracellular effectors from this class [56]. The Hce2 domain of RL4_JR_00199 shares 31% identity and 47% similarity at the amino acid level with the *C. fulvum* Ecp2 effector. The two proteins also share a similar modular architecture and both are small, secreted proteins that contain only the Ecp2 domain. This gene was not differentially expressed in infected redbay trees versus culture in the transcriptomic analysis but a second Hce2-domain-encoding secreted protein of 587 amino acids, RL4_JR_00198, immediately downstream of RL4_JR_00199, is significantly up-regulated during infection (FDR = 2E-18; 5.6 log2 fold change). This predicted, secreted protein shares 35% identity and 50% similarity across the Hce2 domain of *C. fulvum* and is orthologous to Rsp272_RL_00013 in *R. aguacate.*

Additional predicted secreted proteins associated with pathogenicity in other fungi or postulated here to play a role in virulence were found to be unique to *R. lauricola* relative to *R. aguacate* (Supplemental Table S4). In addition to the putative cerato-platanin-encoding gene (RL4_JR_05745) described above, one gene encoding an oxalate decarboxylase (RL4_JR_02745) (FDR = 6E-67; 9.4 log2 fold change) and another gene (RL4_JR_08480) encoding a putative aerolysin (FDR = 7E-72; 6.4 log2 fold change) are up-regulated during infection. Sequence homologs for this predicted aerolysin-like protein, were found in *Ophiostoma piceae* (1E-155)*, Grosmannia clavigera* (1E-143)*,* twenty *Colletotrichum* spp. (6E-133 – 1E-90), a handful of other fungi (3E-82 – 4E-39), and numerous fish species (>6E-11). Comparative structural modeling of this *R. lauricola* protein confirmed a structure consistent with numerous aerolysin-like proteins known to function as toxins or defensive molecules by specific sugar binding through the lectin domain and membrane pore-forming activity of the natterin-like domains of homo-oligomers. The highest scoring structural alignment (protein structural similarity TM-score = 0.757) and overlay is shown in Fig. 1.

An additional ten secreted protein-encoding genes unique to *R. lauricola* and up-regulated (log2 > 1; FDR < 0.05) during infection lacked significant homologs or matched hypothetical proteins in the GenBank non-redundant protein sequence database. These too are considered candidate virulence factors (Supplemental Table S4). Many additional secreted proteins unique to *R. lauricola* relative to *R. aguacate* with predicted roles in host-pathogen interactions however, are also present but not up-regulated during infection. These include four CFEM-domain proteins unique among the 14 total predicted from the genome (Supplemental Table S6). Members of this family include Pth11 known to function in host surface sensing and infection structure development [57]. Of the four unique CFEM proteins one was down-regulated during plant infection and the other three were not differentially expressed between culture and infected plant. Two unrelated secreted proteins with BLASTP homology to “infection structure specific” proteins were also unique to *R. lauricola* (one additional “infection structure specific” protein is shared with *R. aguacate*) but neither gene was up-regulated during infection.

Several predicted secreted proteins with putative plant virulence function annotations that do share an ortholog in *R. aguacate* were also up-regulated during infection (Supplemental Table S7). These include two homologs of the *Pyricularia oryzae* ‘biotrophy associated secreted 2’ proteins (RL4_JR_02461 and RL4_JR_02274; FDR = 7E-4 and 2E-38, respectively; 3.2 and 6.7 log2 fold change, respectively), three homologs of the *P. oryzae* Magnaporthe Appressorium Specific (MAS) protein (GEgh16 homologs; RL4_JR_09303, RL4_JR_02203 and RL4_JR_09408; FDR = 2E-184, 7E-35 and 1E-5 respectively; 7.6, 3.8 and 2.0 log2 fold change, respectively), one ‘infection structure specific’ encoding gene (RL4_JR_03922; FDR = 8E-20; 4.8 log2 fold change) with significant homologs in *Fusarium, Pyricularia, Colletotrichum* and other Ophiostomatales species, and one ‘small secreted’ protein (RL4_JR_06930) remarkable for its significant up-regulation during infection (FDR = 2E-130; 7.8 log2 fold change) and presence of closest homologs in *Ophiostoma piceae* (6E-81) and *Phaeoacremonium minimum* (9E-74) and more than 40 *Fusarium* and *Colletotrichun* species (7E-72 – 1E-62). Although not unique to the pathogen genome, we consider these putative effectors as well.

### Expansion of GH18 and LysM protein domains in *R. lauricola*

**Fig. 3:**
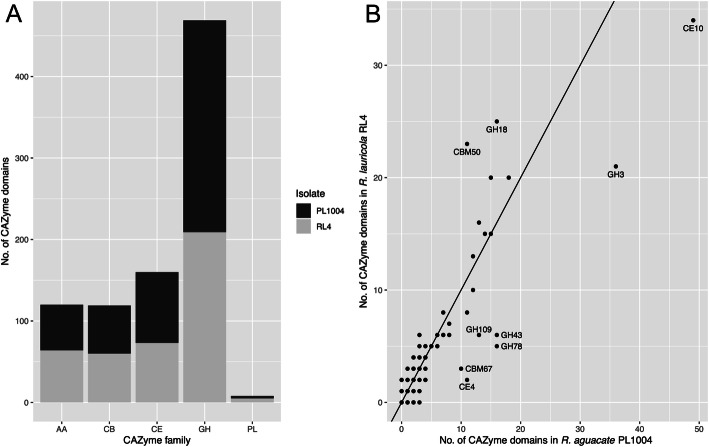
Comparison of the number of Carbohydrate-Active Enzyme (CAZyme) modules across the *R. lauricola* and *R. aguacate* genomes. **a** Occurrence of CAZyme families (AA, CB, CE, GH, PL) within each genome. **b** Enrichment of CAZyme domains between the two genomes. AA: Auxiliary Activities; CB: Carbohydrate-Binding: CE: Carbohydrate Esterases; GH: Glycoside Hydrolases; PL: Polysaccharide Lyases

**Fig. 4:**
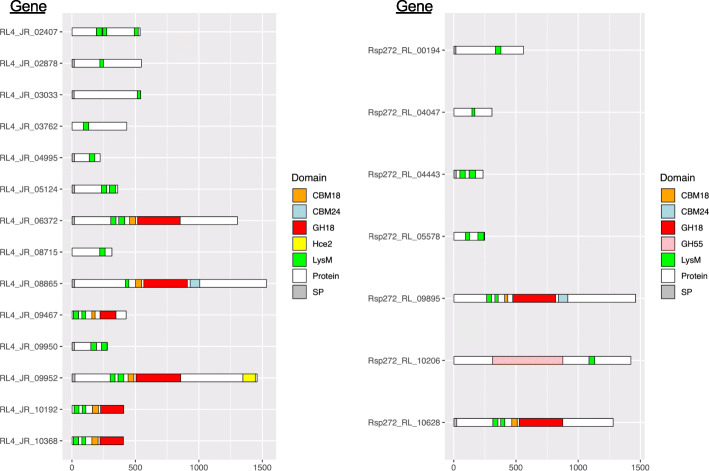
Domain organization of LysM- (CBM50-) containing proteins between the two *Raffaelea* genomes. CBM18: chitin binding 1 domain (PF00187); CBM24: α-1,3-glucan-binding; GH18: chitinase (PF00187); GH55: pectate lyase 3 domains (PF12708); Hce2: pathogen effector, putative necrosis-inducing factor (PF14856); LysM (CBM50): chitin binding (PF01476); SP: signal peptide

A genome-wide comparison of encoded carbohydrate-active enzymes (CAZymes) between the *R. lauricola* and *R. aguacate* genomes was performed. We searched the two *Raffaelea* genomes using the dbCAN Web server (http://www.cazy.org), and compared their inventories. For each CAZyme class, the number of CAZyme domains and their family assignments are shown in Supplemental Table S8 and Fig. 3 The genomes of *R. lauricola* RL4, and *R. aguacate* PL1004 encode a total of 448 and 495 CAZyme domains, respectively. Overall, the two *Raffaelea* species possess similar numbers of CAZyme modules in most CAZyme classes; the glycosyl hydrolase (GH) family is the most prevalent CAZyme family and the polysaccharide lyase (PL) is the smallest CAZyme family among those distributed across both *Raffaelea* genomes (Fig. 3a). Seven CAZyme domains were found to be expanded in the non-pathogenic *R. aguacate* PL1004 relative to *R. lauricola* RL4 (Fig. 3b). This enrichment was for the carbohydrate binding module (CBM) domain CBM67 (ʟ-rhamnose binding), carbohydrate esterase (CE) CE4 domain (de-acetylases), CE10 domain (carboxylesterases), the glycosyl hydrolase (GH) GH3 domain (broad substrate specificity exo-acting sidases), the GH43 (α-L-arabinofuranosidases, α-L-arabinanases, and β-D-xylosidases), the GH75 (β − 1,4-chitosanases), and the GH109 (α-*N*-acetylgalactosaminidase) domain (Fig. 3b). *R. lauricola* RL4 on the other hand, is enriched for two families, both related to chitin. Twenty-five GH18 (chitinases) domains are encoded by *R. lauricola* relative to 16 encoded by *R. aguacate* and twenty-six *R. lauricola* CBM50 (chitin binding; LysM) domains are predicted, more than twice the number (eleven) predicted from *R. aguacate* (Fig. 3b). In *R. lauricola* these 23 LysM domains are encoded by 14 genes whereas the 11 LysM domains from *R. aguacate* are encoded by seven genes (Fig. 4). Among the *R. lauricola* proteins, five encode LysM domain-only secreted proteins while only two *R. aguacate* genes encode LysM domain-only proteins. Among the other LysM-containing proteins from both genomes, functional domains including α-1,3-glucan-binding (CBM24), chitin binding_1 domains (CBM18), chitinase (GH18) and pectate lyase_3 domains (GH55) are present but their functions remain to be investigated.

### Secondary metabolism gene clusters are expanded in the *R. lauricola* genome

The chemical product and function of most fungal secondary metabolite clusters (SMC) are unknown but the prediction and comparative analysis of these biosynthetic pathways is a strong starting point for identifying putative toxin biosynthesis genes. For this reason, the SMCs of the two *Raffaelea* genomes were predicted using two independent programs, SMURF [58] and the antiSMASH webserver [59]. Due to the differences in the algorithms used by SMURF and antiSMASH, the two programs identified overlapping but not identical SMC genes. To obtain a comprehensive list of putative SMCs, we combine the common and unique predictions of SMCs from both predictions (Table 2).

**Table 2:** Summarized SMURF and anti-SMASH results for two *Raffaelea* genomes

	Type	*R. lauricola* RL4	*R. aguacate* PL1004
SMURF	NRPS	3	2
	PKS	12	8
	PKS-NRPS	5	0
	Terpene	0	0
	Other	7	6
	Total	27	16
Antismash	NRPS	4	2
	PKS	11	7
	PKS-NRPS	7	1
	Terpene	7	9
	Other	6	7
	Total	35	26
**Merged**	**NRPS**	**4**	**2**
	**PKS**	**11**	**7**
	**PKS-NRPS**	**7**	**1**
	**Terpene**	**7**	**9**
	**Other**	**8**	**8**
	**Total**	**37**	**27**

**Fig. 5:**
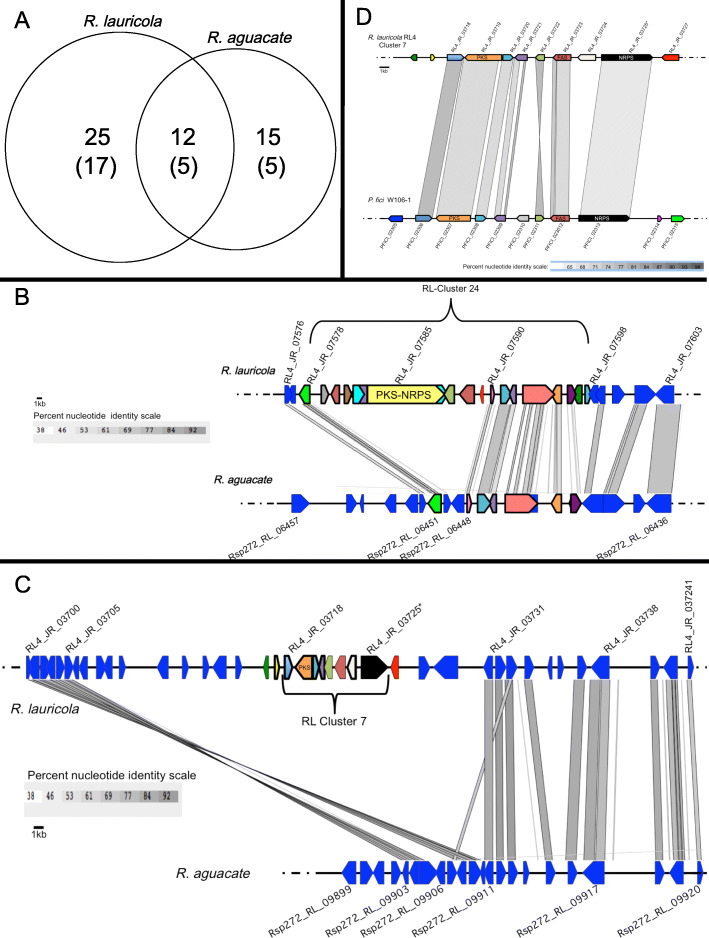
Secondary metabolite gene clusters in *Raffaelea* species. **a** Venn diagram showing distribution of secondary metabolic gene clusters between the two *Raffaelea* genomes. Total numbers of non-ribosomal peptide synthetase (NRPS), polyketide synthase (PKS), and PKS-NRPS gene clusters are shown in parenthesis. **b** Synteny analysis between *R. lauricola* secondary metabolite gene cluster 24 and *R. aguacate*. **c** Synteny analysis between *R. lauricola* secondary metabolite gene cluster 7 and *R. aguacate*. *An overlapping, convergently transcribed gene (RL4_JR_03726) is also predicted on the opposite strand but not shown. **d** Synteny analysis between *R. lauricola* secondary metabolite gene cluster 7 and *Pestalotiopsis fici*

*R. lauricola* is predicted to encode a total of 37 secondary metabolism clusters relative to the 27 predicted clusters in *R. aguacate*. (Fig. 5a). Details of cluster key enzyme genes and accessory genes are given in Supplemental Table S9 and S10. Utilizing synteny and sequence homology as guides, 11 SMC were determined to be shared between *R. lauricola* and *R. aguacate* (four PKSs, one NRPS, three Terpenoids, three Other products)*.* Thus, *R. lauricola* contains 26 unique SMCs (seven PKSs, three NRPSs, seven PKS-NRPSs, four Terpenoids, five Other products) and *R. aguacate* encodes 16 unique SMCs (three PKSs, one NRPS, one PKS-NRPS, six Terpenoids, five Other products). This analysis indicates that there are more than twice the combined number of NRPSs, PKSs and PKS-NRPS hybrids in *R. lauricola* relative to *R. aguacate* (22 vs10). Notably, *R. lauricola* RL4 is predicted to encode seven PKS–NRPS hybrids, whereas non-pathogenic species *R. aguacate* PL1004 encodes a single unique PKS–NRPS hybrid. Taken together, these data reflect an increased potential for secondary metabolite production in the pathogenic *R. lauricola*.

Layering transcriptomic data onto the SMC data determined that 10 of the *R. lauricola* SMC key enzymes (Clusters 7, 8, 11, 16, 17, 18, 22, 24, 25, and 32) are up-regulated *in planta* compared to in vitro. Of these, cluster 7 (NRPS) and cluster 24 (PKS-NRPS) are not found in the *R. aguacate* genome (Supplemental Tables S9 and S10). The anchoring PKS of Cluster 24 shares homology with other PKSs including the *Ophiostoma piceae* UAMH 11346 PKS but no further synteny with clustered accessory genes was found. A comparison of the genomic regions up and downstream of this missing cluster in the *R. aguacate* genome indicated that the PKS-encoding gene as well a seven genes up- and four genes down-stream of this key enzyme gene were absent from the *R. aguacate* genome or present in dispersed, non-clustered regions of the genome (Fig. 5b). Cluster 7 represents an even more extreme example of an insertion or deletion event in which not only were the eight genes predicted for Cluster 7 missing from the syntenic region of the *R. aguacate* genome, but also an additional 11 genes up- and five genes down-stream of the cluster were absent (Fig. 5c). Examining Cluster 7 in more detail determined that this cluster shares a significant level of homology and synteny with an uncharacterized cluster from *Pestalotiopsis fici* W106–1 (Fig. 5d) but not with other SMCs from other species. This cluster is notable for the presence not only of the anchoring NRPS but also a fatty acid synthase (acyl-synthetase) and a second NRPS (“HC-toxin synthetase”).

Of the remaining *in planta* differentially expressed key enzyme clusters, Clusters 16 and 17 appear to encode siderophores (in addition to Clusters 6 and 9 which are not significantly expressed *in planta*) and Cluster 8 appears to encode at least a partial dihydroxynapthelene-based melanin biosynthetic pathway (a PKS with high homology to other melanin PKSs and a clustered putative *tetrahydroxynaphthalene reductase).* The remaining up-regulated *in planta* clusters (Clusters 11, 18, 22, 25, and 32) that are found in both *R. lauricola* and *R. aguacate* are predicted to encode polyketides (3 clusters), an undefined pathway (1 cluster) and a terpenoid product (1 cluster) (Supplemental Table S9).

### Comparative transcriptomic analysis

In addition to cataloging putative virulence associated genes through comparative genomics we also took a non-biased transcriptomics approach to identify genes differentially expressed during plant infection relative to growth in culture. For this comparative transcriptomic analysis, three biological replicates of RNA extracted from *R. lauricola* grown on solid growth medium and from *R. lauricola*-inoculated redbay trees were used. Two genotypes of redbay, one considered fully susceptible (‘HIE’) and the other considered tolerant (‘HIL’) were utilized. To confirm the host responses of the two redbay genotypes, laurel wilt disease scores were assessed following trunk inoculation. At 60 days post inoculation, the average disease score for ‘HIE’ was 5 with a mortality rate of 100% and was 3 for ‘HIL’ with a mortality rate of 20%, (Supplemental Table S11). Hence, ‘HIE’ was considered to be a laurel wilt susceptible genotype and ‘HIL’ was considered to be a tolerant genotype.

When mapping reads of the 21 RNAseq samples (6 water-inoculated stems, 6 *R. lauricola*-inoculated stems, 6 distal leaf samples, 3 in vitro-grown *R. lauricola* cultures) to the *R. lauricola* genome assembly, only the six fungal-inoculated stem tissue samples (three ‘HIE’ and three ‘HIL’) and the three in vitro-grown cultures contained fungal reads and were further analyzed. The percentage of fungal reads in the plant inoculated samples ranged from 0.4% (170,662) to 2.9% (972,592) of the total read pairs (Supplemental Table S12). From the in vitro-grown fungal cultures, approximately 91 M reads per replicate were retained after read cleaning and adaptor removal for further alignment. No fungal genes were found to be uniquely up- or down-regulated between the two redbay genotypes (data not shown). Subsequent analysis therefore, compared the in vitro grown culture treatment against the two plant-inoculated treatments as six biological replicates of a single treatment, i.e., plant infection. The analysis of redbay differentially-regulated transcripts will be reported in a separate publication.

**Fig. 6:**
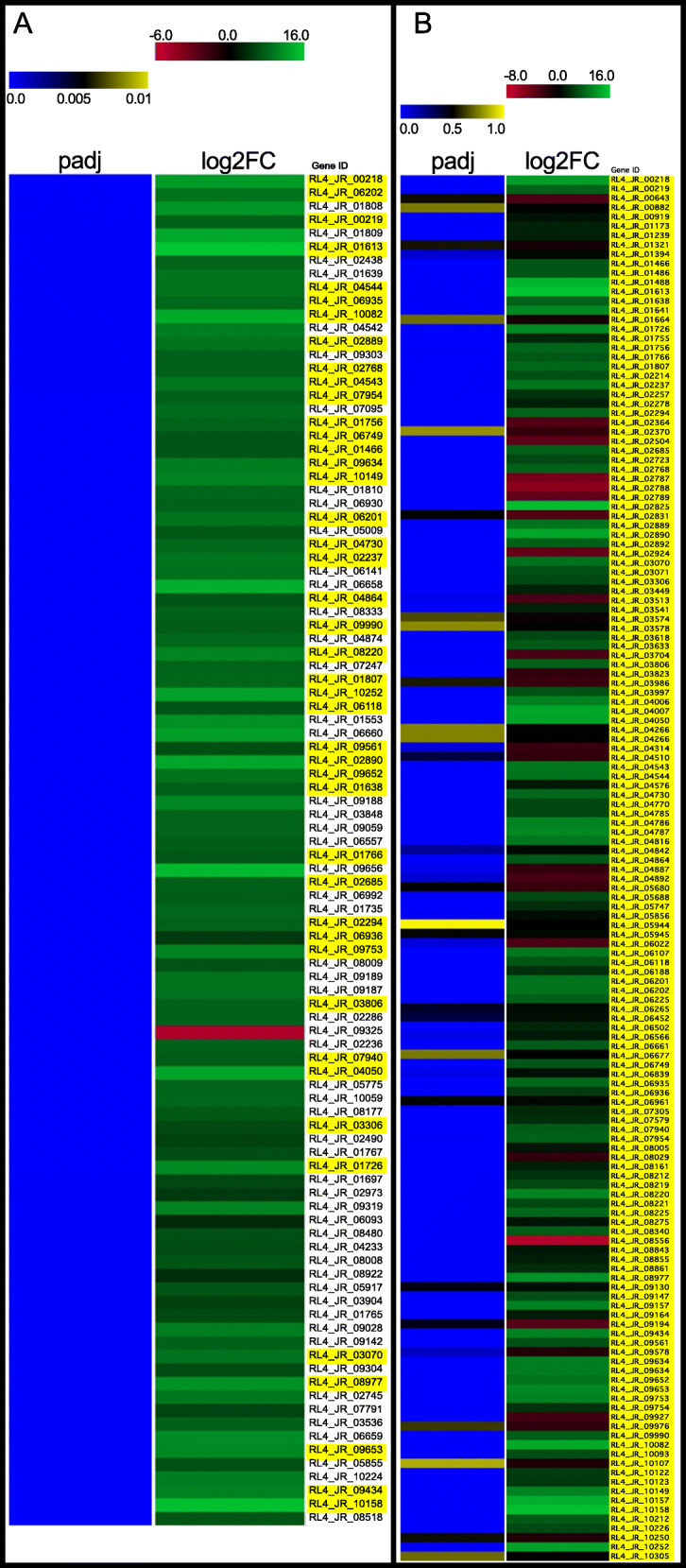
Heatmaps of differentially expressed *R. lauricola* genes represented as log2 fold change (log2FC) from infected redbay versus in vitro culture. **a** Top 100 differentially expressed genes sorted by adjusted *P* values (padj) from smallest to largest. **b** Differential expression of 150 genes annotated for roles in alternative sulfur uptake or assimilation sort by gene ID. Gene IDs highlighted in yellow are annotated for involvement in alternative sulfur uptake or assimilation

**Fig. 7:**
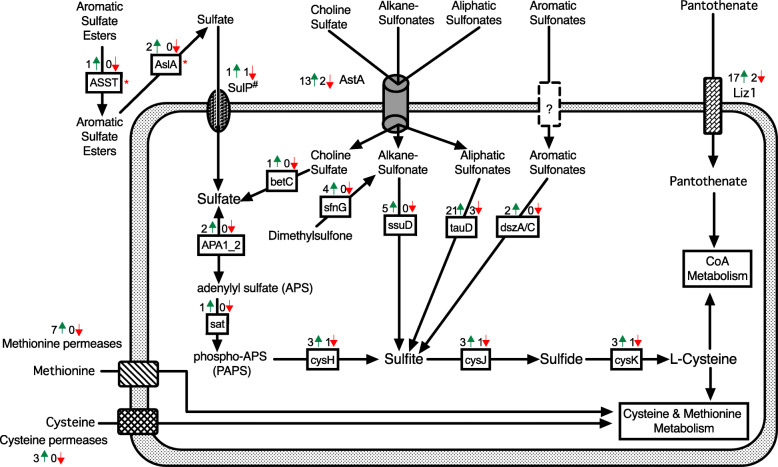
Model of *R. lauricola* alternative sulfur uptake and assimilation pathways based on differential gene expression. The number of genes up-regulated (green arrow) or down-regulated (red arrow) from each enzyme or transporter family is indicated. Enzyme and transporter abbreviations are given in Supplemental Table S13. ^#^SulP: Sulfate permease superfamily 2.A.53; Sulfate permease II 2.A.53.1.2 *Neurospora crassa* Cys-14 homolog is up-regulated (5.8 log2), a putative sulfate permease (Sul1; 2A.53.1.11) is down regulated (− 1.9 log2) and three additional unlikely sulfur transporter family members are listed in Supplemental Table S13

When comparing gene expression from in vitro grown cultures to the inoculated redbay stems, 4679 (2070 up-regulated and 2609 down-regulated) differentially expressed genes (DEGs) were obtained (FDR < 0.05; |log2 fold change| > 1) (Supplemental Table S7). Differential regulation data for many genes with putative roles in pathogenicity have been presented in the preceding sections of these results. Taking a more global view of the data, we see that within the top 100 differentially regulated genes (sorted adjusted *P*-value), 44 are alternative sulfur source (sulfides, sulfoxides, sulfones, sulfonates, sulfate esters and sulfamates) uptake or assimilation related genes (Fig. 6a and Supplemental Table S7). At the genome level, genes encoding homologs of known sulfur transporters including sulfate permeases, high affinity methionine and cysteine permases, and alternative sulfate transporters, as well as enzymes functioning in desulfurization of organosulfur compounds including predicted extracellular and intracellular taurine dioxygenases, arylsulfatases, arylsulfotransferases and alkanesulfonate monooxygenase are abundantly encoded within the *R. lauricola* genome. In total, we annotated 150 sulfur source uptake or assimilation-related genes based on transporter annotation and Enzyme Commission classification (Supplemental Tables S13 and S14). Of these sulfur-related genes, 108 (72%) are up-regulated *in planta*, 19 (13%) are down-regulated, and 23 (15%) exhibit no significant change (FDR < 0.05; |log2 fold change| > 1) (Fig. 6b). To summarize this data, a putative metabolic pathway for predicted *R. lauricola* genes encoding alternative sulfur source uptake and assimilation with the total number of predicted genes up- or down-regulated for each gene is shown in Fig. 7 and more detailed analysis of their classifications and differential expression is provided below.

Several classes of sulfur compound transporters were annotated as Transporter Classification Database Family Members (TCDFM). The four main groups include methionine permeases, cysteine permeases, sulfate permeases, and alternative sulfur transporter family members (Supplemental Table S13). All seven of the high affinity methionine permeases (TCDFM: 2.A.3.8.4) and all three of the high affinity cysteine permeases (TCDFM: 2.A.1.14.20) are all up-regulated during plant infection (average 6.8 log2fold up-regulation). One putative sulfate permease (TCDFM: 2.A.53.1.2/SulP) sharing strong homology to the Cys-13 and Cys-14 sulfate permeases of *N. crassa* is up-regulated during infection, but the *N. crassa* Sul1 homolog is down-regulated. Three additional SulP-related family members (TCDFM: 2.A.53.1.11/.7/.8) of unknown function or putative sodium bicarbonate transport are all down-regulated. Thirteen of fifteen alternative sulfur transporter family members (TCDFM: 2.A.1.14.38/ AstA) are up-regulated during infection. Additionally, although not a direct sulfur compound transporter, 17 of 19 pantothenate family transporters (2.A.1.14.17 TCDFM) are up-regulated during plant infection. Pantothenic acid (vitamin B5) is required to synthesize the sulfur-containing coenzyme-A (CoA). Other annotated sulfur-related transporters and their differential regulation profiles during plant infection are listed in supplemental Table S13.

Regarding assimilation of alternative sulfur sources, genes encoding enzymes to metabolize diverse sets of organic sulfur compounds including aliphatic, aromatic and alkane sulfonates, choline sulfate and aromatic sulfur esters are massively up-regulated during infection (Supplemental Table S14). For example, the diversity and scale of α-ketoglutarate-dependent sulfonate dioxygenase-encoding gene family members (PFAM: TauD/TfdA-like domain; InterPro entry IPR003819) includes 27 members with an average 6 log2fold up-regulation (64x). In addition to these aliphatic sulfonate dioxygenases, five alkane sulfonate monooxygenase-encoding genes are predicted and all five are up-regulated during infection (average 8.1 log2fold up-regulation (274x)). Additional organic sulfur assimilation enzyme-encoding genes are also up-regulated. The transcript accumulation dynamics of these genes is shown in Supplemental Table S14. Although there are unique genes without corresponding orthologs in many of these sulfur assimilation enzyme families in *R. lauricola* relative to *R. aguacate*, *R. aguacate* also encodes many unique family members lacking orthologs in *R. lauricola* (Supplemental Table 14). Thus, neither species appears to be enriched in sulfur assimilation genes relative to the other. When comparing the relative abundance of the TauD domain among other Sordariomycetes species, *Neurospora crassa* encodes 13 TauD family members, *Podospora anserine* 23, *Colletotrichum graminicola* 18, *C. higginsianum* 28, *Beauveria bassiana* 22, *Metarhizium anisopliae* 11, *Verticillium dahliae* 13, *V. longisporum* 22, Nectria haemoatococca 26, *Neonectria ditissima* 28, *Pestalotiopsis fici* 37, *Sporothrix schenckii* 42, *Ophiostoma piceae* 27 and *Ceratocystis platani* 7 (http://pfam.xfam.org) [60]. From this sampling, no clear pattern relating TauD protein domain abundance with tree pathogens or insect associations is evident.

Genes encoding transporters and assimilation enzymes for the uptake and utilization of alternative sulfur sources in filamentous fungi are known to be positively regulated by the global bZip transcription factor Cys-3 in *Neurospora crassa* (the MetR ortholog in *Aspergillus* spp.) and the negative regulator Scon-2. The Cys-3 ortholog was identified by BLASTP homology and RSD analysis of the *R. lauricola* predicted proteins. The ortholog is structurally misannotated and encompasses two gene IDs (RL4_JR_07387 and RL4_JR_07388) in the current predicted gene calls. Of these RL4_JR_07388 is significantly (FDR = 8.3E-18) up-regulated during plant infection (2.0 log2fold up-regulation (2.6x)) and RL4_JR_07387 is slightly up-regulated (0.7 log2fold; FDR = 0.02; Supplemental Table S15). An ortholog of the Scon-2 negative regulator (RL4_JR_01708) was also identified from the predicted gene calls. In *N. crassa* Scon-2 is known to be positively regulated by Cys-3 and, consistent with this known regulatory model, it is up-regulated during plant infection in the *R. lauricola* transcriptome analysis (1.8 log2fold up-regulation (3.5x); FDR = 8.9E-9; Supplemental Table S15). On the basis of homology to known *N. crassa* alternative sulfur source regulators, annotations of these and other putative regulators are shown in Supplemental Table S15.

## Discussion

Ambrosia beetles and their fungal symbionts generally colonize dead or dying host trees and thus historically were not considered a major threat to healthy tree ecosystems [32]. Laurel wilt, however, has been recognized as an emerging disease since 2004 and fits the previously proposed new-encounter model in which native but not exotic host species tolerate infection by ambrosia beetle symbionts [33]. The ability of ambrosia beetles and their fungal symbionts to colonize living trees in their native habitats in fact has been suggested to have utility in pre-invasion evaluations to identify potential tree-killing invasive pests [18]. A major challenge is to understand what factors have driven the evolution of a symptomatically mild disease in the native range and yet results in a lethal, tree-killing disease in a newly invaded environment. With both native and non-native hosts, *R. lauricola* faces hostile conditions as the beetles delivers its spores into a living tree with intact defense mechanisms. Pathogenicity traits may allow these fungi to survive and proliferate in the unique ecological niche of the living trees’ xylem vessels by initially overcoming or avoiding the host immune system. Subsequently, when conidia have been distributed within the xylem, the pathogen may deploy virulence mechanisms leading to wilting and increased colonization. When introduced to new, closely-related but not sympatrically co-evolved hosts, this balance of attack and defense is tilted in favor of the pathogen with its novel virulence mechanisms and a host population lacking the corresponding counter defense mechanisms. The work presented here has pursued the hypothesis that *R. lauricola* is an adapted pathogen on its native hosts in Asia and that these adaptations may be uncovered through comparative genomics. Several novel *R. lauricola* genes have been identified in support of this hypothesis and provide a starting point to understand how the fungus induces symptoms and poses such a serious threat to avocado, redbay and other members of the Lauraceae family in the western hemisphere. A potential virulence role for several of these genes is supported by transcriptomic data. The failure to uncover pathogen transcripts differentially regulated between the susceptible and tolerant redbay genotypes may be due to a lack of statistical power resulting from to the relatively low level of fungal transcripts recovered from infected trees. Conversely, the fungal infection processes, reflected in gene expression, may not differ significantly between the two plant genotypes at the chosen sampling stage. More refined sampling in future studies to include additional sampling points and methods to enrich for fungal transcripts should provide a more comprehensive view of both pathogen and host processes occurring in susceptible and tolerant host interactions.

The annotation and comparative genomic resources established here for the laurel wilt pathogen *R. lauricola* and the closely related non-pathogenic *R. aguacate* provide the starting material for functional gene analysis. Vanderpool et al. [37] previously reported assemblies for these two species from Illumina HiSeq reads resulting in assemblies of 207 and 414 scaffolds for *R. lauricola* and *R. aguacate*, respectively (GenBank assembly accession: GCA_002778145.1 and GCA_002777955.1). Ibarra Caballero et al., [40] also published a draft genome of *R. lauricola* isolate C2646 from NextSeq reads resulting in an assembly of 1535 scaffolds (GenBank assembly accession: GCA_004153705.1). The draft genomes reported in this current work improves upon these previous assemblies resulting in 169 and 368 scaffolds, respectively. The current work develops these genomic resources further by providing gene calls from the new assembly, providing detailed annotation of comparative gene content between the two species, and analyzing the transcript accumulation dynamics of the *R. lauricola* genes during host infection.

Comparison of predicted secretomes of the two *Raffaelea* species indicated the presence of secreted protein genes unique to *R. lauricola* RL4. Notably, a cerato-platanin gene homolog was among the pathogen-unique secreted protein genes. Cerato-platanins act as plant immunity elicitors and may function as necrotrophic effectors or pathogen–associated molecular patterns (PAMPs) [61]. That this well-known plant immunity elicitors exists in *R. lauricola* RL4 but not in its non-pathogenic relative *R. aguacate* PL1004, leads to the simple hypothesis that secretion of cerato-platanin by *R. lauricola* during its colonization of avocado triggers a hypersensitive host defense response that contributes to wilt symptom development. The role of cerato-platanins in pathogenesis however varies among pathogens from contributors to necrosis and host defense responses, to no apparent effect on pathogenicity [52, 62]. In addition to other characterizations of the *R. lauricola* cerato-platanin protein, loss-of-function mutants and over-expression strains for the certao-platanin gene are currently under development to determine its role in laurel wilt disease.

Another gene unique to *R. lauricola* and highly expressed during plant infection is RL4_JR_08480, a gene encoding an aerolysin-like protein. The lectin domains of aerolysin-like proteins have been demonstrated to provide binding specificity within lipid membranes triggering oligomerization of the natterin domain which inserts into the lipid bilayer to allow electrolyte leakage and disrupt membrane function. Members of this family are pore-forming proteins first described as a pore-forming toxin from the bacterium *Aeromonas hydrophila* [63]. In numerous bacteria where they have been characterized they function to kill host cells or other competing bacterial cells [64]. Aerolysin-like proteins are now known to be encoded by organisms as phylogenetically diverse as fungi, plants and animals [65]. In many vertebrate and invertebrate animals, they are thought to play defensive roles against potential pathogens but their role in plant pathogenesis has not been characterized [66, 67]. Three characteristics of the *R. lauricola* aerolysin-like protein support the hypothesis that this protein plays a role in *R. lauricola* virulence: (i) it is a secreted protein that exhibits very strong structural homology to other known aerolysin proteins that function in membrane pore formation, (ii) relative to *R. aguacate*, it is unique to *R. lauricola* and other pathogenic fungi, and (iii) transcripts encoding the RL-aerolysin are strongly and significantly up-regulated during plant infection. More detailed transcript and protein profiling in the host and beetle mycangia coupled with the creation and characterization of gene-specific deletion mutants are planned to test its function.

Besides the cerato-platanin and aerolysin-like genes, there are several other genes encoding small, secreted proteins with homology to known virulence factors unique to *R. lauricola.* These include seven genes encoding Hce2-domain proteins, whereas its non-pathogenic relative *R. aguacate* PL1004 only possesses one. All Hce2 proteins contain the Ecp2 domain, and based on protein domains and sequence length, they can be grouped in three classes: class I contains small secreted proteins of 80–400 amino acid (aa); class II proteins contain the modular architecture similar to class I proteins, but they are much longer (up to 800 aa); class III proteins contain a composite modular architecture with the Ecp2 domain fused to the C-terminus of fungal subgroup GH18 chitinases [55]. Among the seven *R. lauricola* Hce-2 genes, only RL4_JR_00199 (155 aa) matches the known features of an extracellular effector (class I). Although RL4_JR_00199 only shares 30% identity at the amino acid level with the 165 aa *C. fulvum* Ecp2 effector, the similar modular architecture between RL4_JR_00199 and *C. fulvum* Ecp2 effector suggests that the two proteins have an analogous role in plant pathogenesis. Stergiopoulos et al. [68] suggested that *C. fulvum* Ecp2 only weakly perturbs its virulence target without inducing necrosis. However, the Ecp2 effector of *Mycosphaerella fijiensis*, causal agent of the devastating black Sigatoka disease of banana, causes host necrosis and promotes virulence much stronger than the *C. fulvum* Ecp2 [68]. The differences in the virulence functions of Ecp2 effectors from two pathogens has been suggested to reflect the co-evolution of pathogens and their hosts: the hemibiotroph *M. fijiensis* Ecp2 can induce necrosis but the biotroph *C. fulvum* Ecp2 can facilitate pathogen infection without inducing necrosis [68]. Similarly, *R. lauricola*-host interaction in Asia may have fine-tuned Ecp2 activity to only weakly perturb and cause damage to the native Lauraceae trees in Asia without inducing host wilt mortality during their co-evolution. In contrast, due to the lack of co-evolution of *R. lauricola* and Lauraceae species in North America, counter defense to Ecp2 action may not exist and it and other putative effectors potentially contribute to the wilt mortality. Functional analysis of this gene should be performed to test this hypothesis.

Several other predicted, secreted proteins, both unique and shared with *R. aguacate*, are encoded and in many instances demonstrated to be up-regulated during infection. These too, may function as effectors. Repeat sequences in some fungal plant pathogens have been demonstrated to contribute to the divergence and emergence of novel virulence traits among closely related species [49, 69]. Characterization of total repeat content of the two genomes indicated that LTR retroelements are ten-fold more abundant in the *R. lauricola* genome. Whether this increase is associated with variation in effector or other pathogenicity-related genes awaits the functional identification of such traits.

Besides toxins, secondary metabolism clusters (SMCs) are known to produce many compounds with potential roles in fungal development and ecology or to function as effectors within host cells perturbing cell signaling and altering cell morphology [70]. In agreement with the analysis conducted by Ibarra Caballero et al., [40] comparative analysis of SMCs here demonstrated that the *R. lauricola* possess significantly more SMCs than the non-pathogenic species. The difference is most striking for NRPSs, PKSs, and hybrid PKS-NRPS, important enzyme families involved in toxin biosynthesis [70]. The significantly larger number of PKS and NRPS gene clusters in pathogenic *Raffaelea* species is consistent with its expanded capacity for pathogenesis. The expanded array of PKS-NRPS hybrid clusters in *R. lauricola* relative to *R. aguacate* (7 versus 1) is of particular note. Previous investigations on the PKS-NRPS hybrids from the rice blast fungus *Magnaporthe grisea* and the fungal biocontrol agent *Trichoderma* spp. showed that the PKS-NRPS hybrid can mediate pathogen recognition and induce plant defense responses [71, 72] . Despite the higher number of SMCs present in *R. lauricola*, only two unique clusters were found to be up-regulated *in planta* based on transcriptomic data. These clusters are predicted to encode a NRPS product and a PKS-NRPS product. These clusters represent putative toxin biosynthetic clusters but does not fully explain the lack of expression of other unique clusters *in planta*. The relative low abundance of fungal transcripts present in the sampled tissue as well as the limited sampling of the interaction may account for the inability to get an accurate assessment of differential gene expression for all clusters. This lack of resolution is inherent to the interaction in which the pathogen is detected at low biomass and the large woody nature of the host makes it difficult to spatially sample the host-pathogen interaction. Other SMCs unique to RL may be of further interest including Cluster 4 which, based on homology and synteny with *Fusarium* spp., appears to encode the pathway for a polyketide related to fusaric acid.

The infection of *R. lauricola* induces the formation of gels and tyloses in xylem lumena [28], which is a common plant defense response functioning to close off xylem vessels and lock out invading vascular pathogens [73–75]. The strategy of deploying virulence factors that elicit plant defense responses such as tyloses and gums leading to physiological malfunction, wilting, and host mortality at the appropriate time, balanced with the avoidance of recognition early in the interaction is common with necrotrophic and vascular wilt pathogens [76–78]. As such, *R. lauricola*, in addition to offensive effectors, is expected to have masking effectors to avoid the triggering of PAMP-triggered immunity (PTI) from chitin or other PAMPs.

Chitin is a major constituent of fungal cell walls, and its fragments, chitin oligosaccharides, are well-documented PAMPs [79]. Several plant chitin receptors located in the plasma membrane have been identified. These receptors contain extracellular LysM domains [80, 81]. Fungi too produce exracellular LysM domain proteins to sequester chitin oligomers and block host recognition. Predominantly, fungal LysM proteins can be classified into two groups. In the first group LysMs are associated with chitinase domains (GH18). The second group contains secreted LysM effector proteins, e.g. Ecp6, that possess multiple LysMs but no catalytic domains, [82]. *R. lauricola* encodes members of both groups indicating that it has the ability to hydrolyze chitin as well as a means for protecting its own chitin from degradation and host recognition. Interestingly, LysM effectors are not pathogen-specific and they occur in both pathogenic and non-pathogenic fungi [83]. These LysM proteins may also help mutualistic symbiotic microbes, endophytes and other microbes to establish intimate relationships with their hosts. In pathogens, it has been shown in the fungal tomato leaf mold pathogen *C. fulvum* that the LysM effector Ecp6 displays a significant higher chitin-binding affinity than that of plant immune receptors, and it can prevent fungal cell wall–derived chitin fragments from being perceived by host immune receptors, and thus perturb host immunity [84]. Furthermore, two wheat blotch pathogen, *Mycosphaerella graminicola*, LysM effectors were demonstrated to not only block the elicitation of chitin-induced plant defenses, but also prevent fungal hyphal lysis by plant hydrolytic enzymes [85]. Thus, fungal LysM effectors may play diverse roles during host colonization. CAZyme analysis with the two *Raffaelea* genomes reported here determined that *R. lauricola* possess a significant expansion of LysM domains relative to *R. aguacate* PL1004. The remarkable difference between the number of LysM domains in pathogenic and non-pathogenic *Raffaelea* species elicits speculation that in its native southeastern Asia range, coevolution of *R. lauricola* and native Lauraceae trees involves a continuous arms race, in which *R. lauricola* LysM effectors have duplicated and diversified to avoid host immunity responses to a point where the fungus may deploy virulence factors (e.g., cerato-platanin, aerolysin-like protein, Hce2) when it is spatially and temporally poised to take advantage of the host resources. This hypothesis may explain why there are no reports of laurel wilt causing mortality on native Lauraceae trees in Asia in that the balance of pathogen recognition and avoidance of recognition has led to non-lethal disease symptomology. On the contrary, due to the lack of coevolution of *R. lauricola* and Lauraceae trees in the southeastern United States, the LysM effectors may be so effective in avoiding host recognition that a buildup in pathogen colonization is unchecked allowing the deployment of other effectors and elicitors leading to the over-stimulation of host defense systems and host physiological malfunction. Functional analysis of *Raffaelea* LysM proteins, especially the pathogen-specific secreted LysM proteins would provide a test of this hypothesis and better define the roles of LysM proteins in ambrosial fungi during plant host colonization as well as gallery and mycangial biome dynamics.

In addition to the secretion of proteins and metabolites for establishing compatibility and promoting disease, pathogens must also adapt their metabolism to that of the host environment. The transcriptomic analysis presented here suggests that *R. lauricola* experiences sulfur starvation during infection of its host. Inorganic sulfur is an essential nutrient likely to be available in very limited supply during xylem colonization. Although avocado fruit are rich in sulfur, primarily glutathione, the sulfur makeup in other organs of the plant are not well characterized. In general, sulfur is taken up by plant roots in the form of sulfate in the xylem. Glutathione may also load in tree root-mychorrizal associations and transport systemically through the phloem [86]. Thus the availability of sulfur within the xylem is thought to be primarily or exclusively in the form of inorganic sulfate. Alternative sulfur uptake and assimilation pathways in filamentous fungi are positively regulated by the absence of elemental and amino acid-based sulfur sources [87]. The extreme up-regulation of the alternative sulfur uptake and assimilation genes in *R. lauricola* implies that inorganic sulfate is extremely limited in the xylem and other tissue colonized by *R. lauricola* during infection necessitating the pathogen to scavenge sulfur from alternative, organic sulfur sources.

Organic forms of sulfur within the xylem of healthy trees may include the phenolic intermediates of lignin biosysnthesis including *p*-coumaroyl-CoA, caffeoyl-CoA and feruloyl-CoA [88] as well as xenobiotic sulfur compounds that may accumulate as defense compounds [89–91]. Both elemental sulfur [91] and organic sulfur metabolites e.g., glucosinolates [89] are known to play roles in plant defense against fungal pathogens. The organic sulfur content of tree species, particularly their vascular tissues, is not extensively documented but studies of sulfur content of Norway spruce identified spectra consistent with organic sulfate and sulfate esters as major forms of sulfur accumulated in latewood annual rings [92]. In addition, in beech trees, sulfate esters were the dominant form of sulfur accumulated in woody tissue [93]. Thus varying sources of organic sulfur are present in woody tissues of tree species that, in the absence of sulfate or other common forms of organic sulfur including methionine, may serve as essential sources of sulfur for colonizing fungi. The competition between the host and the pathogen for these organic forms of sulfur is likely to be high during disease development. Whether *R. lauricola* is reacting to sulfur starvation conditions or sulfur starvation conditions coupled with sulfur-based defense is not readily apparent. What is apparent is that *R. lauricola* has the genetic capacity to utilize a diverse range of organic sulfur compounds including aromatic sulfonates and sulfate esters as well as aliphatic and alkane sulfonates. This is strongly supported by gene content and differentially regulation during infection. The ability to transport and assimilate these alternative organic sulfur sources may function as a competitive advantage for *R. lauricola*. This hypothesis is being pursued through characterization of the *R. lauricola* Cys-3 homolog which is known to function as a positive global regulator of the alternative sulfur assimilation pathways in Ascomycota fungi.

## Conclusions

While *R. lauricola* is presumed to have originated in Asia and introduced to North America around 2002, *R. aguacate* PL1004 was isolated from a dead avocado tree in Miami-Dade County, Florida in 2009 [35] and has been isolated subsequently from the mycangia of *Xyleborus bispinatus* collected from avocado trees [94]. *X. bispinatus* is an endemic ambrosia beetle species that can transmit *R. lauricola* to avocado in Florida [95]. *R. aguacate* has been determined to be non-pathogenic on all hosts that have been screened [96]. Thus, *R. aguacate* and *R. lauricola* share similar morphology, can be associated with the same ambrosia beetle species, are both recovered from avocado, but differ in their pathogenic potential. Given the similarities in biology between them, comparative genomic analysis presented here provides evidence for the enrichment of traits related to adapted pathogenesis in the laurel wilt pathogen. These include evolved strategies to avoid or combat defense mechanisms of living trees and the capacity to secrete putative effectors, such as cerato-platanin, an aerolysin-like protein and Hce2, and to produce SMCs to potentially induce necrosis and elicit host defense responses. It also encodes the ability to adapt its metabolism to the host xylem. During the co-evolution of *R. lauricola* pathogenicity and Lauraceae tree resistance in Asia, a more balanced host response may dampen symptomology. Due to the lack of arms race co-evolution of the invasive *R. lauricola* pathogen and the Lauraceae family in the southeastern USA, plant hosts appear to fail in restricting pathogen spread, allowing virulence factors from the pathogen to promote colonization and induce a strong host response including the occlusion of xylem vessels by tyloses and gel and phenolic compound accumulation leading to the host mortality. Evidence from these comparative analyses and from transcriptomic analysis of *R. lauricola-*inoculated redbay trees provides support that *R. lauricola* is an adapted pathogen in Asia and the lethal symptomology observed in North America is due to an evolutionary mismatch resulting from a lack of co-evolution between the host and pathogen in the western hemisphere.

## Methods

### Genome sequencing and assembly

Single-end sequencing of the genomes of *R. lauricola* RL4 (originally collected from a laurel wilt diseased avocado (*P. americana*) tree in 2009 on Merritt Island, FL, USA; CBS-KNAW collection number: 127349) [97] and *R. aguacate* PL1004 (originally collected from an avocado tree in 2009 from Miami-Dade county, FL, USA; CBS-KNAW collection number: 141672) [35] was performed on the Ion Torrent Personnel Genome machine (PGM) by the Interdisciplinary Center for Biotechnology Research (ICBR) Genomics Core at the University of Florida. Ion torrent reads were assembled using the de Bruijn algorithm implemented in the CLC Genomics Workbench version 5.0.1 (CLC Bio, Aarhus, Denmark), MIRA 4 [98], and Spades 3.7.1 [99]. Contig N50 value and total contig number were used to decide the best assembly. The *R. lauricola* RL4 and *R. aguacate* PL1004 genomes were also sequenced using Illumina Hiseq technology from one pair-end (100 bp) and two mate-pair (100 bp and 250 bp) libraries. The Illumina reads were assembled using the ALLPATHS-LG assembler [38]. Assemblies generated from Ion Torrent and Illumina reads were merged using Metassembler [39] to achieve a potentially better final assembly. These Whole Genome Shotgun projects have been deposited at DDBJ/ENA/GenBank under accessions JACBXF000000000 and JACCPH000000000. The versions described in this paper are versions JACBXF010000000 and JACCPH010000000.

### Repeat content

RepeatModeler 1.0.8 (http://www.repeatmasker.org/RepeatModeler.html) and RepeatMasker 4.0.6 (http://www.repeatmasker.org/) were used to perform the repetitive element analysis. In brief, RepeatModeler, which uses RepeatScout and RECON [100, 101] de novo repeat library algorithms, was used to generate de novo repetitive element predictions for the *Raffaelea* genomes using the RMBlast NCBI search engine. The generated de novo repetitive element predictions and fungal-specific repetitive element libraries in the RepBase database (http://www.girinst.org/repbase/index.html) were subsequently searched to identify and categorize repetitive elements.

### Transcriptome assembly for gene modeling

Total RNA was extracted from liquid culture grown *R. lauricola* RL4 and *R. aguacate* PL1004. Sequencing was performed with Illumina HiSeq 2000. We assembled the RNA-Seq reads into transcripts using genome-guided and de novo RNA-Seq assembly approaches using Trinity 2.2.0 [41]. Using the de novo and genome-guided transcriptome assemblies as input, a comprehensive transcriptome database was generated using the PASA pipeline [42]. Genome-guided and de novo assembly was done using Trinity with the “–genome_guided_max_intron 3000” option and the default setting for genome-guided and de novo assembly, respectively. PASA pipeline was run under the default setting.

### Gene prediction and annotation

Genes of *Raffaelea spp.* were predicted by two cycles of Maker pipeline using the reviewed proteins in Uniprot database as the protein evidence, and transcripts from PASA pipeline as additional EST evidence for gene prediction. RepeatMasker (http://www.repeatmasker.org) was used to mask the repeats in the genome sequence based on repetitive fungal sequences from RepBase [102] and repetitive sequences identified by RepeatModeler (http://www.repeatmasker.org/RepeatModeler.html). In the first cycle of Maker, only GeneMark-ES v.4.32 was used. Gene modules predicted by Maker with a strong annotation quality score (AED = 0) were used for the SNAP training and Augustus training. In the second cycle of Maker, Augustus, GeneMark, and SNAP were employed for gene prediction. To assess the validity of the final assembly and gene prediction, Benchmarking Universal Single-Copy Orthologs (BUSCO) was used to provide an estimate of assembly and annotation completeness [47].

NCBI’s non-redundant (nr) protein database was used for BLASTP queries of all predicted gene models to obtain annotation descriptors in addition to InterProScan5 classifications [103] and KEGG GhostKOALA (https://www.kegg.jp/ghostkoala/). Gene models were also annotated by Blast2GO v4.0 [104] to derive a list of top annotated BLASTP hits. Secretomes of each fungal species were first predicted using SignalP 4.1 [105], candidates containing transmembrane helices predicted by TMHMM 2.0 [106] were removed. Genes encoding putative carbohydrate-active enzymes were identified by using the dbCAN Web server (http://csbl.bmb.uga.edu/dbCAN/blast.php) for automated CAZy annotation [107]. Sulfur uptake and assimilation related genes were derived from genes in the KEGG sulfur metabolism reference pathway (https://www.genome.jp/kegg-bin/show_pathway?map=map00920andshow_description=show) and sulfonate transporter genes were identified by BLASTP homology from the published work of Holt et al. [108].

Transporters were predicted by querying the Transporter Classification Database http://www.tcdb.org/ by BLASTP using the *R. lauricola* predicted proteins. Enzymes associated with utilization of alternative sulfur sources were mapped to the KEGG reference map for sulfur metabolism using a combination of EC numbers, InterPro IDs and BLASTP homology. Enzyme names were retained from the reference pathway or the closest filamentous fungal homolog where available and described more fully in Supplemental Table S13. Effectors were predicted from the *R. lauricola* predicted secreted proteins using EffectorP 2.0 [48]. Hmmsearch in the HMMER3 package was used to identify Raffaelea proteins containing the Hce2 domain (Pfam: PF14856). CAZy domain information (LysM, CBM18, CBM24, GH18, and GH55) of Raffaelea proteins was extracted from CAZy annotation outputs of dbCAN Web server. The R package gggenes (available at https://github.com/wilkox/gggenes) was used to provide graphical representations of protein domain architecture.

Tertiary structure prediction of secreted proteins RL4_JR_08480 (aerolysin-like) and RL4_JR_05745 (cerato-platanin) were modeled on the I-TASSER server [109]. Within I-TASSER, the highest scoring model was utilized to identify proteins with structural similarity within the Protein Data Bank (PDB) [110] using TM-align [111]). Structural matches with the highest TM-score are presented. Structural models were viewed and images downloaded for presentation from the NCBI iCn3D web-based 3D structure viewer [112]. The 3M3G cerato-platanin protein structure was obtained from the crystal structure of the *Trichodema virens* Sm1protein (DOI: 10.2210/pdb3M3G/pdb). The Dnl1 aerolysin-like protein dimer was obtained from the solved 1.86 Å crystal structure from zebrafish (**DOI:** 10.2210/pdb4ZNO/pdb; Jia et al., 2016). The identification of common and species-specific genes between the two *Raffaelea* species was performed using the Reciprocal Smallest Distance (RSD) method [113].

### Secondary metabolic gene cluster analysis

The secondary metabolic gene clusters (SMCs) were identified using the Secondary Metabolite Unknown Region Finder (SMURF) web-based program [58] and the antiSMASH pipeline [59]. Non-redundant clusters from both predictions were combined and manually annotated via BlastP.

### Plants and growth conditions

Two clonally propagated genotypes (HIE and HIL) of redbay trees (*Persae borbonia*) were used. These clones were obtained from the native ecosystem on Hunting Island South Carolina under South Carolina State Parks Research Permit N-06-08 and identified as *P. borbonia* by Dr. Marc Hughes and confirmed through microsatellite analysis by Katherine Smith (USDA-Forest Service). Voucher specimens are housed at the University of Florida Herbarium. These genotypes, known to vary in their susceptibility to laurel wilt [23], were grown in five-gallon containers (4–5 years old) with a dominant stem 2–2.5 cm in diameter and 1–1.5 m in height. Plants were maintained in the greenhouse with 16-h light and 8-h dark under ambient temperature conditions (average temperature ranged from 60 °F to 80 °F). Trees were irrigated daily and fertilized as needed. To confirm the categories of susceptible and tolerant, laurel wilt disease scores were assessed following trunk inoculation with wild-type isolate RL4. At 60 days post inoculation, the average disease score for HIL was 3 with a mortality rate of 20%, and 5 for HIE with a mortality rate of 100% (Table S11). Hence, HIE was considered to be a laurel wilt susceptible genotype and HIL was considered to be a tolerant genotype.

### Fungal cultures for inoculum production and in vitro growth

*Raffaelea lauricola* isolate PL571 (GenBank JQ861956.1) was revived from its glycerol stock by streaking on cycloheximide-streptomycin malt agar (CSMA) [114]. Cultures were incubated at room temperature (approximately 23 °C) for seven days. Approximately 10 ml of sterilized water was added to the surface of the plates and a spreader was used to agitate the surface gently. The suspension was collected by pipette and spore concentration was measured using a haemacytometer. The final suspension was diluted to 1 × 10^6^ spores per ml. For in vitro cultures utilized in the RNA-Seq analysis, a spore suspension of *R. lauricola* isolate PL571 was spread on the surface of a 9 cm cellophane-covered PDA (potato dextrose agar) culture plate and incubated for seven days under ambient laboratory conditions (approximately 23 °C; 10 h light). Fungal tissues (spores and hyphae) from three separate biological replicates were harvested into 1.5 ml microtubes, lyophilized overnight and stored at − 80 °C until RNA extraction.

### Redbay tree inoculations for disease ratings and RNA-Seq tissue sampling

For redbay trunk inoculations, two 2-mm-diameter holes, 1 cm apart, were drilled into each side of the main stem (7.5 mm deep) at a 45° angle, 15–30 cm above the soil line. Approximately 50 μl of an *R. lauricola* isolate PL571 conidial suspension (ca. 5 × 10^4^) or water (control) was pipetted into each hole. The inoculation sites were sealed with Parafilm. For all inoculation assays, the four treatments consisted of HIE and HIL genotypes inoculated with water and HIE and HIL genotypes inoculated with *R. lauricola* spore suspension. For RNA-Seq analysis, three days after inoculation, three inoculated trees of each treatment (12 trees in total) were selected for sample collection. For each tree, the stem encompassing the inoculation sites (5 mm above the upper hole and 5 mm below the lower hole) and 9 distal leaves 3 each from 3 independent branches were collected. Samples were immediately placed into liquid nitrogen and then stored at − 80 °C until further processing for RNA extraction.

To confirm the unpublished susceptible (HIE) and tolerant (HIL) phenotypes under our greenhouse conditions, five additional trees were rated for laurel wilt disease for each of the four treatments as described above at 60 days post inoculation (20 trees in total). Laurel wilt disease ratings were scored according to the method of Hughes et al. [30]. The tree inoculation and laurel wilt disease scoring experiment was repeated once and the ranking of the HIE genotype as susceptible and the HIL genotype as tolerant was confirmed.

### RNA purification and sample preparation for RNA sequencing

Tree tissue samples were ground in liquid nitrogen using a bead beater and Lysing Matrix A (MP Biomedicals LLC, Solon, OH). Approximately 50 mg of each ground sample was used for RNA extraction using the method of Chang et al. [115]. The RNA was further treated with DNAase and concentrated using RNA Clean and Concentrator-5 (Zymo Research). RNA samples were submitted to GENEWIZ for library preparation and RNA sequencing (Illumina HiSeq3000, 2 × 150 bp, with PolyA Selection). For in vitro-grown fungal tissue, approximately 10 mg of the freeze-dried fungal tissue for each sample was homogenized using a bead beater. Total RNA was extracted using the RNeasy Plant Mini kit according to the manufacturer’s instruction (QIAGEN). The Interdisciplinary Center for Biotechnology Research (ICBR) NextGen DNA Sequencing core, University of Florida (UF) performed mRNA isolation using NEBNext Ploy(A) mRNA Magnetic Isolation module (New England Biolabs, catalog # E7490) and RNA library construction with NEBNext Ultra RNA Library Prep Kit for Illumina (New England Biolabs, catalog # E7530) according to the manufacturer’s user guide. Paired-end, 2 × 100 cycle sequencing was performed at the ICBR on two lanes of the Illumina HiSeq3000 instrument using the clustering and sequencing reagents provided by Illumina (San Diego, CA, USA).

### RNA-Seq analysis

Initially, rCorrector [116] and a python script (https://github.com/harvardinformatics/TranscriptomeAssemblyTools) were used to clean up erroneous k-mers and unfixable read pairs from the sequence reads. Trim Galore (https://github.com/FelixKrueger/TrimGalore) was used for quality checking and adaptor trimming of all sequence reads. For the inoculated redbay tree samples, plant reads were obtained by mapping all reads to the RL4 genome assembly and retaining the un-mapped reads for Trinity assembly. Trinity [41] with the default parameters was used to obtain the transcriptome for each of the two redbay genotypes. The transcriptome quality was evaluated using BUSCO [47]. Bowtie 2 [117] was used to map the redbay reads to this assembled transcriptome. For fungal transcript mapping, TopHat2 [118] was used to map the in vitro fungal reads and the inoculated redbay tree reads to the RL4 genome assembly. DESeq2 [119] was used for differential gene expression analysis using the default parameters. RNAseq sequence data is available through NCBI under accession PRJNA637370.

## Supplementary information

**Additional file 1: Supplemental Figure S1**. Bioinformatic pipeline utilized for *Raffaelea lauricola* and *R. aguacate* genome assembly and gene prediction.

**Additional file 2: Supplemental Figure S2**. Multiple sequence alignment of *Raffaelea lauricola* putative cerato-platanin protein (RL4_JR_05745) and *Botrytis cinerea* cerato-platanin protein BcSpl1. Arrows indicate conserved cysteine residues.

**Additional file 3: Supplemental Table S1** - Repetitive elements identified within genome assemblies. **Supplementary Table S2**. Predicted effector proteins (EffectorP 2.0) and differential expression for *R. lauricola.* **Supplementary Table S3**. Predicted effector proteins (EffectorP 2.0) for *R. aguacate* PL1004. **Supplemental Table S4**. Predicted secreted proteins unique to *R. lauricola* RL4 relative to *R. aguacate* PL1004. **Supplementary Table S5**. Genes encoding secreted proteins unique to *R. aguacate* PL1004 relative to *R. lauricola* RL4. **Supplemental Table S6**. Master annotation table with RNAseq data. **Supplemental Table S7**. *R. lauricola* differentially expressed genes. **Supplementary Tabe S8**. Number of CAZy family members in two *Raffaelea* genomes. **Supplementary Table S9**. Secondary metabolite cluster genes in *R. lauricola* RL4. **Supplementary Table S10**. Secondary metabolite cluster genes in *R. aguacate* PL1004. **Table S11**. Disease scores of the inoculated redbay trees at 60 days post inoculation. **Table S12**. Read pairs in fungus inoculated stem samples. **Table S13**. *R. lauricola s*ulfur transporters. **Table S14**. *R. lauricola* and *R. aguacate s*ulfur assimilation genes. Table S15. *R. lauricola* alternative sulfur regualtory genes.

## Data Availability

The Whole Genome Shotgun projects have been deposited at DDBJ/ENA/GenBank under Bioproject PRJNA635322 accessions JACBXF000000000 (*R. lauricola* RL4) and JACCPH000000000 (*R. aguacate* PL1004). The versions described in this paper are versions JACBXF010000000 and JACCPH010000000. Genome assemblies, predicted genes and proteins and mapping files are also available from the Dryad Dataset at 10.5061/dryad.05qfttf14. The RNAseq datasets generated in the current study are available in the NCBI repository under accession PRJNA637370. Other RNAseq datasets utilized during gene prediction in the current study are available in the GenBank repository under accession numbers SRX3033598 and SRX3033591. Other data sets or sequences utilized in this work can be found in NCBI under the following accessions: JQ861956.1, GCA_002778145.1, GCA_002777955.1, SRX3033598, SRX3033591 and GCA_004153705.1.

## Abbreviations

*antiSMASH*: Antibiotics and secondary metabolite analysis shell
BLASTP: Basic local alignment search tool protein
BUSCO: Benchmarking Universal Single-Copy Orthologs
CAZymes: Carbohydrate-active enzymes
CBM50: Carbohydrate-binding module 50
CE: Carbohydrate esterase
CFEM: Cysteine-containing fungal extracellular membrane
CoA: Coenzyme-A
CSMA: Cycloheximide-streptomycin malt agar
DEGs: Differentially expressed genes
EST: Expressed sequence tag
ETI: Effector-triggered-immunity
FDR: False discovery rate
GEgh: Gene Erysiphe graminis forma specialis hordei
GH: Glycoside hydrolase
Hce2: Homologs of *C. fulvum* Ecp2 effectorHR
HIE: Hunting Island clone E
HIL: Hunting Island clone L
HR: Hypersensitive responses.
ICBR: Interdisciplinary center for biotechnology research
*KEGG*: *Kyoto encyclopedia of genes and genomes*
LINE: Long interspersed nuclear element
LTR: Long terminal Repeat
LysM: Lysin motif
MAS: Magnaporthe Appressorium Specific
MAT: Mating type locus
Mb: Mega base
NCBI: *National Center for Biotechnology Information*
NPP1: Necrosis inducing protein
NRPS: Non-ribosomal peptide synthetase
PAMP: Pathogen-associated molecular pattern
PASA: Program to Assemble Spliced Alignments
PDA: Potato dextrose agar
PDB: Protein data bank
PGM: Personnel genome machine
PKS: Polyketide synthase
PL: Polysaccharide lyase
PRRs: Pattern recognition receptors
PTI: PAMP-triggered immunity
Q20: Sequence quality score of 99%
RMBlast: Repeat masker basic local alignment search tool
RNA-Seq: Ribonucleotide nucleic acid sequencing
SMC: Secondary metabolite clusters
SMURF: Secondary metabolite unique regions finder
*SNAP*: Semi-HMM-based nucleic acid parser
SulP: Sulfate permease
TauD: *Taurine* dioxygenase
TCDFM: Transporter classification database family members
TM-score: Template modeling score
