# Supplementary figures and images for: Genomic and transcriptomic insights into Raffaelea lauricola pathogenesis
Source: BMC Genomics. 2020 Aug 20;21:570. doi: 10.1186/s12864-020-06988-y (PMC7441637; doi:10.1186/s12864-020-06988-y)

**Fig. S1**

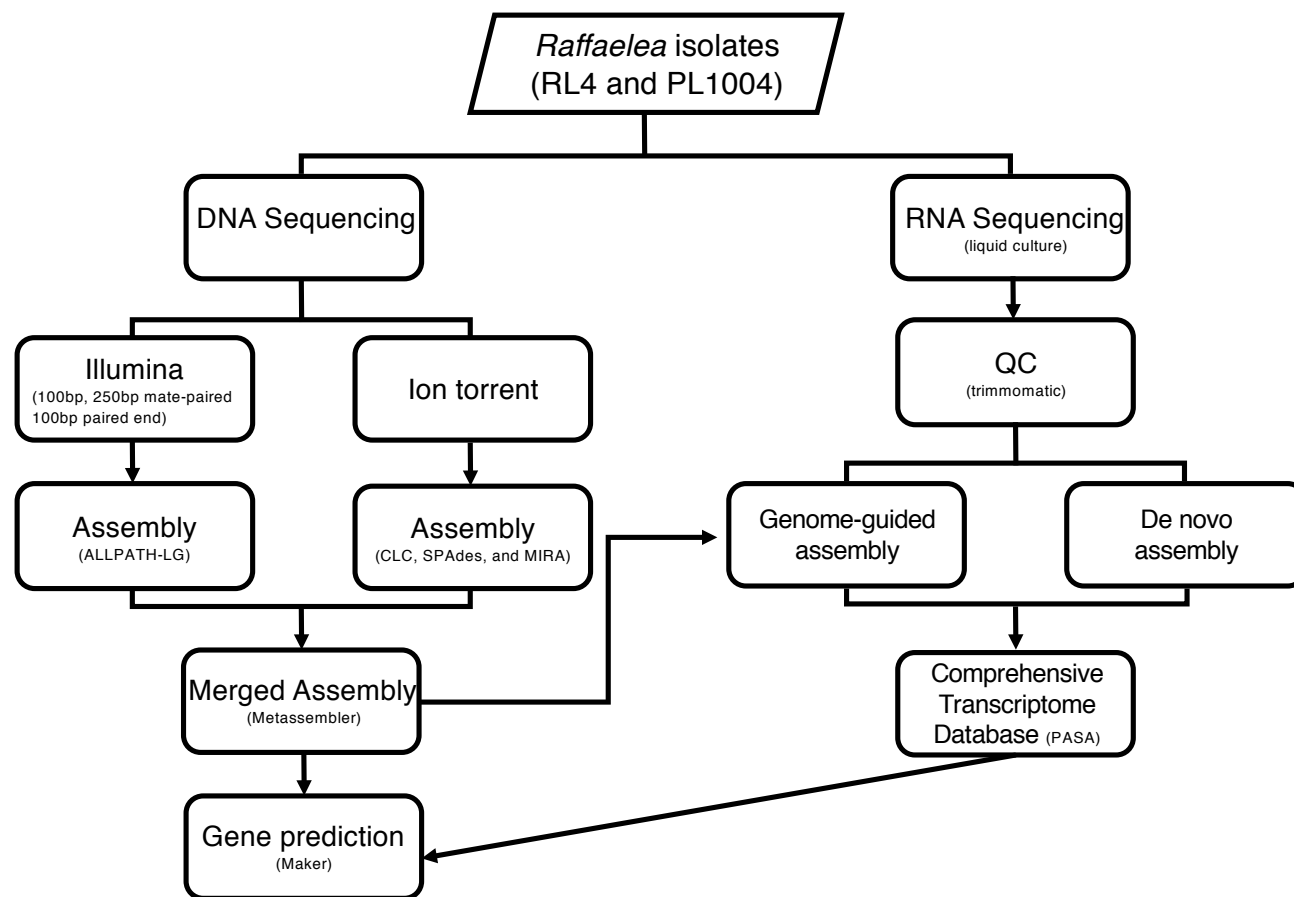

Supplement: Supplementary file 1 — Additional file 1: Supplemental Figure S1. Bioinformatic pipeline utilized for Raffaelea lauricola and R. aguacate genome assembly and gene prediction. [file 12864_2020_6988_MOESM1_ESM.pdf]

Fig. S2

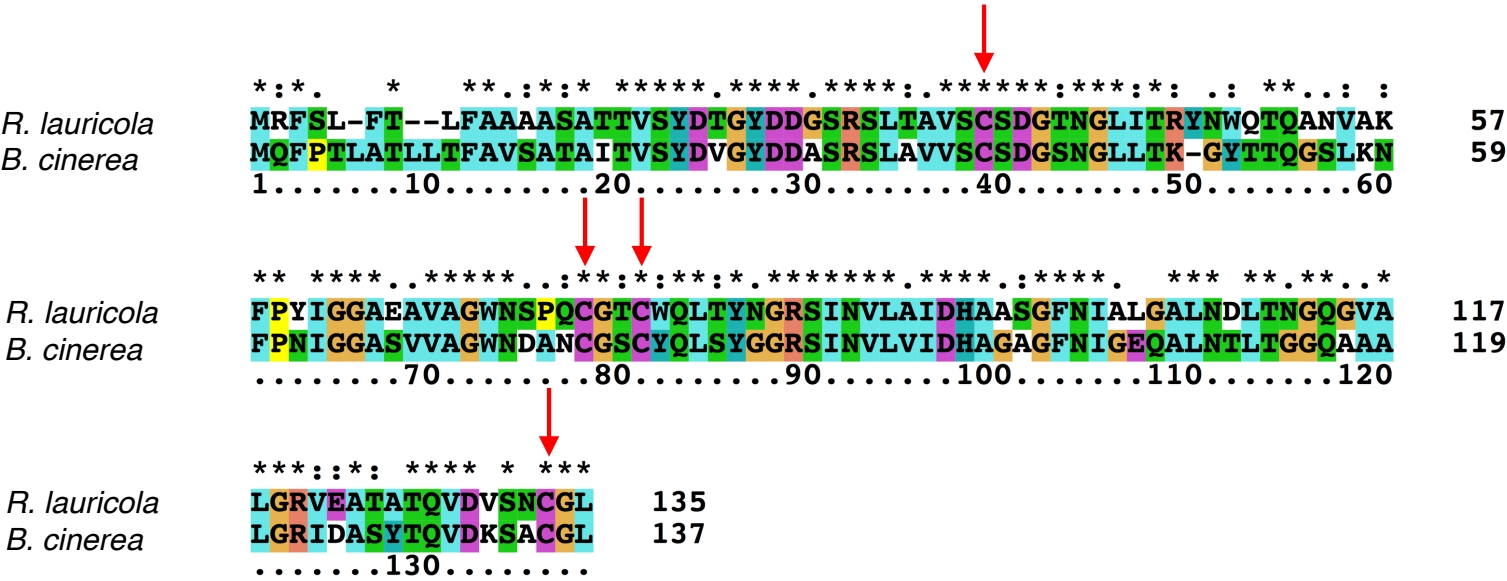

Supplement: Supplementary file 2 — Additional file 2: Supplemental Figure S2. Multiple sequence alignment of Raffaelea lauricola putative cerato-platanin protein (RL4_JR_05745) and Botrytis cinerea cerato-platanin protein BcSpl1. Arrows indicate conserved cysteine residues. [file 12864_2020_6988_MOESM2_ESM.pdf]
